# Design and implementation of a terahertz lens-antenna for a photonic integrated circuits based THz systems

**DOI:** 10.1038/s41598-022-05338-0

**Published:** 2022-01-27

**Authors:** Shihab Al-Daffaie, Alaa Jabbar Jumaah, Verónica Laín Rubio, Thomas Kusserow

**Affiliations:** 1 Terahertz Nanophotonics and Integration Technology (TNIT), Center for Photonic Science and Engineering, Skolkovo Institute of Science and Technology (Skoltech), Moscow, Russian Federation 121205; 2 Institute of Microwave Engineering and Photonics, Technical University of Darmstadt, 64283 Darmstadt, Germany; 3 Institute of Nanostructure Technologies and Analytics, University of Kassel, 34127 Kassel, Germany

**Keywords:** Electrical and electronic engineering, Electronic devices

## Abstract

A new integrated lens-antenna is designed and implemented for a nanocontact based terahertz (THz) photomixer. The new design replaces the standard conventional bulky silicon lens, which normally no THz photomixer can avoid. The Fresnel Zone Plate is used to design the new lens-antenna and is simulated by the MIT open-source tool called Meep. The final design showed, with only two simple fabrication technology processing steps (standard optical lithography) that the lens-antenna can be monolithically integrated with the THz nanophotomixer. With its compact design, the THz measurements showed a comparable behavior to the conventional bulky silicon lens, therefore it would be ready for photonic integrated circuits based THz systems.

## Introduction

**Figure 1:**
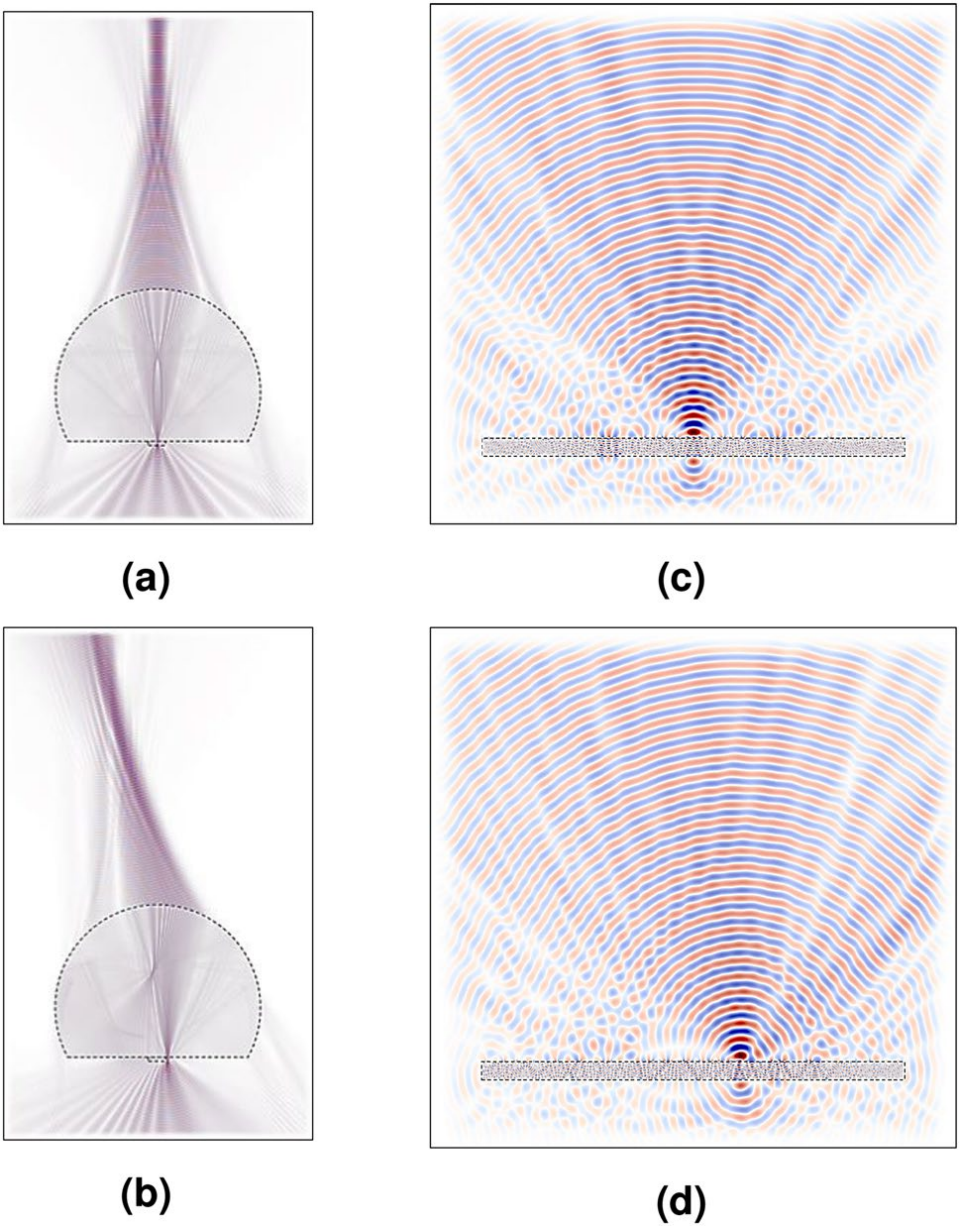
The simulated electric field spread where (**a**) the source is placed at the center of the Si-lens. (**b**) The source is misplaced by 1 mm from the center of the Si-lens. (**c**) The source is placed at the optimal position in the FZP’s center. (**d**) The source is misplaced by 1 mm from the FZP center.

Conventional photomixers use optically illuminated electrodes on a high-speed photo-conductive material, such as low-temperature-grown-GaAs (LTG-GaAs) for THz generation^1^. The generated carriers between the electrodes are transferred and coupled to a suitable antenna for THz radiation. Currently available based THz systems have already demonstrated great potential in terms of high tunability, standard room temperature operation, and signal quality, however they are still suffering from many drawbacks, such as big size equipment (needs an optical table), mechanical disturbance (additional to noise and alignment), high power consumption (electrical and optical), and low flexibility system (each application needs a new setup)^2^. The researcher therefore proposes a new THz system platform, aimed to overcome all the above drawbacks, based on photonic integrated circuits and nanotechnology^3,4^. The target system-on-chip incorporates a fully integrated THz source and detector with increased emission power and sensitivities by using nano-contacts based vertical nanophotomixers with cointegrated electronic and air-interface. Nanocontact photomixers were already reported in^5^ to overcome the failure mechanisms of the conventional photomixer. It shows a large reduction of the capacitance and increase of the photocurrent as compared to conventional photomixers. However, the generated THz signal requires an additional coupling media to increase the directivity due to the big difference in electric permittivity at the interface GaAs-air. Thus, a bulky silicon lens (Si-lens) is used normally in such THz devices so that certain characteristics of interest, like directivity or modifications in phase or amplitude can be achieved easily^6,7^. However, the bulkiness of these kind of lenses does not allow to have militarized and compact sources for portable applications. Moreover, Si-lenses are highly sensitive to misalignments which can easily occur when mounting the photomixer on the lens, as shown in Fig. 1a, where with only a 1 mm misalignment, the focal point changes drastically its position, as shown in Fig. 1b. One of the main objectives here is to achieve better performance of these THz devices by avoiding the bulkiness of the Si-lens.

A Fresnel zone plate has been chosen to be integrated with the photomixer due to its planar structure and directivity characteristics (e.g. the FZP is commonly used in microscopy in X-rays). FZPs have also been used in a compact cavity-backed FZP lens antenna, integrated in low-temperature co-fired ceramic which was proposed to achieve high gain at 270 GHz band^8^. In our design a different approach has been taken towards a fully integrated photomixer lens-antenna. This design will be suitable for the integrated nanophotomixers based on graphene or nanocontacts to improve the performance^9^, as the new design will give a remarkable solution to improve the directivity of the generated THz waves and avoid misalignment caused by placing the source in a place that is not the center of the Si lens, as shown in Fig. 1c and d. This will be described in detail in the 2D Simulation section.

The design and implementation of the integrated lens-antenna will be described and compared with the typically used Si-lens in the following sections.

## Design

The chosen structure to substitute the bulky Si-lens is the FZP, which will be directly integrated in the substrate.

A FZP consists of a set of so called Fresnel zones alternated in opaque and transparent rings. Fresnel zones are designed in a way that the plane wave arriving at the lens will interfere constructively at the odd zones and destructively at the even ones. Therefore, the even numbered zones block the wave in order to maximize the constructive interference, losing half of the power^10^.

Two design equations are needed to implement this type of diffractive lens^10^: the number of zones N and the radii $r_{n}$ of these, which are as follow:

1 $$\begin{aligned}&N = \dfrac{\lambda f}{4 w^{2}} \end{aligned}$$

2 $$\begin{aligned}&r_{n} = n \lambda \left( f + \dfrac{n \lambda }{4} \right) \end{aligned}$$

where $\lambda$ represents the design wavelength, *f* the focal distance, *w* the resolution and *n* the number of the zone. According to the equations, the more resolution wanted, the bigger the FZP will need to be.

The integrated FZP has been designed for 1 THz, which in a substrate of GaAs ($\epsilon _{r}=12.97$) corresponds to a wavelength of $83.3\,\upmu \hbox {m}$.

**Figure 2:**
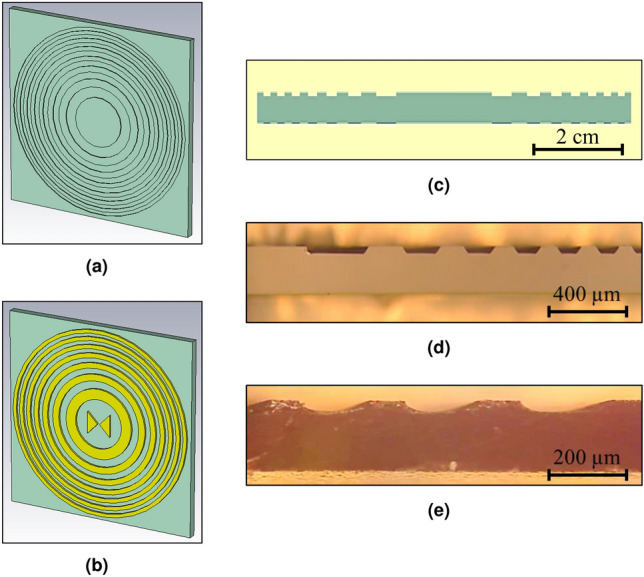
The layout structure and the transversal cut of the lens-antenna device (**a**) the top view. (**b**) The bottom view. (**c**) The transversal cut up the device, where the lens-antenna design was designed for 1 THz with 14 Fresnel zones, a focal plane at 15 mm, and a focal point resolution of 150 μm. (**d**)Transversal cut of the device with anisotropic etching. (**e**) Transversal cut of the device with isotropic etching.

Together with a chosen focal distance of 15 mm and a resolution of 150 μm, a minimum of $N = 14$ Fresnel zones are needed. The calculated radii of Fresnel zones were varied from $r_{1} = 1.1\,\hbox {mm}$ to $r_{14} = 4.2\,\hbox {mm}$, please check "Lens-Antenna Designs" in the supplementary document for more information.

In order to integrate the lens in the device and use all the available power, instead of blocking the even numbered Fresnel zones, the FZP is etched on the surface of the GaAs substrate^11,12^, with an etching depth of $\lambda /2 = 41.65\,\upmu \hbox {m}$. Thus, the wave is phase shifted at the etched level in order to transform the destructive contributions into constructive contributions at the focal point. The result of this etched FZP is shown in Fig. 2a.

Moreover, a set of gold rings with the radii $r_{n}$ is placed around the photomixer and right underneath the etched Fresnel zones of the FZP. These, which are also shown in Fig. 2b, reflect the back radiation, providing better collimation and directivity, please check "Lens-Antenna Designs" in the supplementary document for more information.

In addition, a transversal cut of the device is shown at the bottom of Fig. 2c, where the etched FZP is illustrated on the top of the substrate and the gold rings on the bottom, in a darker blue color. Therefore, the integration and flatness achieved with this design can be appreciated. Figure 2d and e show optical images for two etched samples using two different etching methods, which will be described in detail in the Device Implementation section.

## Simulation results

Two different software programs have been used to simulate the new lens-antenna: Meep (an MIT open-source tool simulation) for 2D simulations and CST Microwave Studio for 3D simulations. The 2D simulation with Meep was precisely addressed the drawback of the misalignment position of the THz source compared to CST Microwave Studio. Meanwhile, the CST Microwave Studio 3D simulation was clearly showed the 3D parameters, like the directivity and the efficiency of the lenses.

### 2D simulations with Meep

**Figure 3:**
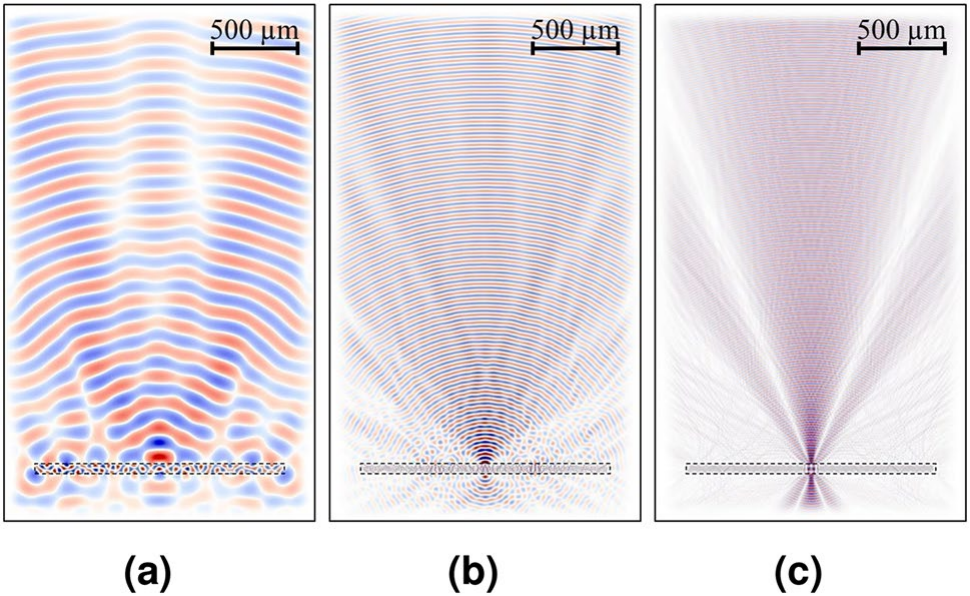
Meep FDTD simulations for (**a**) 300 GHz. (**b**) 1 THz. (**c**) 3 THz. The lens-antenna represented by a dashed rectangle.

The device has been designed and simulated for 1 THz. It is seen from the resulting electric field distribution, that there is propagation out of the GaAs substrate and collimation of the beam towards a focal point shown in Fig. 3b. However, this point appears closer to the device than what was specified in the design parameters ($f = 15\,\hbox {mm}$). This is due to the fact that the source is not placed at the focal point of the corresponding lens or FZP, but right at the bottom of the substrate, which has a thickness of $300\,\upmu \hbox {m}$^13^.

Another important feature of the new integrated lens-antenna is that a wideband (from 300 GHz to 3 THz) was achieved by the simulation and by measurements, as shown in Fig. 3a and c, respectively, and it will be explained in the measurement results section. It is above the bandwidth that can be achieved by the bulky Si-lens. Meep shows that the best result corresponds, as expected, to the design frequency of 1 THz as shown in Fig. 3. The shape of the beam is different for the different frequencies, but the focal point is still achieved.

Moreover, a focal point can be achieved even if there is a misalignment of the photomixer of 1 mm with respect to the center of the FZP as shown in Fig. 1c and d. This would mean a big advantage in integration of the THz devices compared with the Si-lens. Moreover, since a simple fabrication process integrates both the photomixer and the FZP, little misalignments are acceptable.

### CST microwave studio 3D simulations

**Figure 4:**
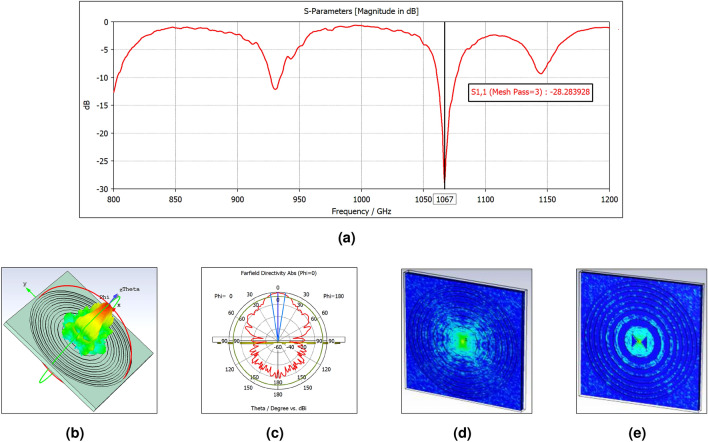
The CST results at 1.067 THz: (**a**) the $\hbox {S}_{11}$ parameter with a minimum at 1.067 THz. (**b**) The 3D far-field. (**c**) The polar chart. (**d**) The electric field distribution for the FZP’s side. (**e**) The electric field distribution for the antenna’s side.

In order to extract more information about the new integrated lens-antenna, additional simulations have been performed with CST Microwave Studio. The S11 parameter is obtained as shown in Fig. 4a, where the minimum is at 1.067 THz.

Taking into account this result, the far field diagrams (3D and polar chart) at this frequency, can be seen in Fig. 4b and c, respectively. A maximum directivity value of 19,9 dBi is reached for a frequency of 1.067 THz with this design. Therefore, not only the size is reduced in all dimensions compared with a common Si-lens, but also a higher directivity is earned.

Moreover, the strong interaction of the electric field with the first Fresnel zone at 1.067 THz is better represented in Fig. 4d and e.

## Device implementation

The device implementation involves only two simple fabrication technology processing steps. In the first step, the photomixer and the gold rings are fabricated by the optical lithography and the gold evaporation. The second lithography is done in the second step before the wet etching of the FZP. The alignment between both top and bottom sides is determined by the dicing of the sample through the alignment marks. This method results in one of the biggest advantages compared with the Si-lens, which is the integration of both the photomixer and the lens monolithically.

**Figure 5:**
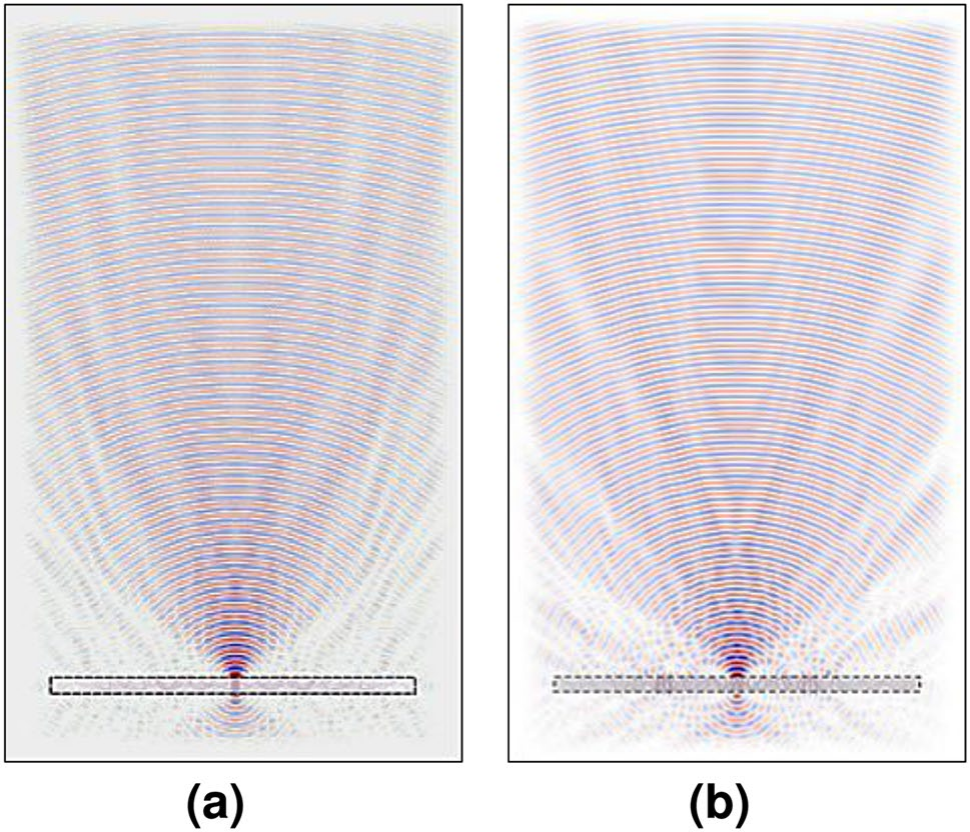
The optical image and the Meep results for 1 THz: (**a**) anisotropic wet etching. (**b**) Isotropic wet etching.

Two different etchings have been tested in different FZPs: $H_{2}O:H_{2}SO_{4}:H_{2}O_{2}$ with the proportions 10 : 1 : 8 (anisotropic wet etching, Fig. 2d) and $H_{3}PO_{4}:H_{2}SO_{2}$ in 1 : 1 (isotropic wet etching, Fig. 2e). A closer look to the transversal cut of the etched FZP shows that the anisotropic etching process generates angled etches in the lens, as can be seen in Fig. 2d. However, according to the Meep 2D simulation results shown in Fig. 5, this will not have a negative effect in neither the propagation nor the directivity of the wave, due to the diffractive nature of the lens. In fact, it slightly improves the performance of the device, closing the beam towards the focal point. This could even be more significant with the isotropic etching, please check "Implementation Process and Results" in the supplementary document for more information.

**Figure 6:**
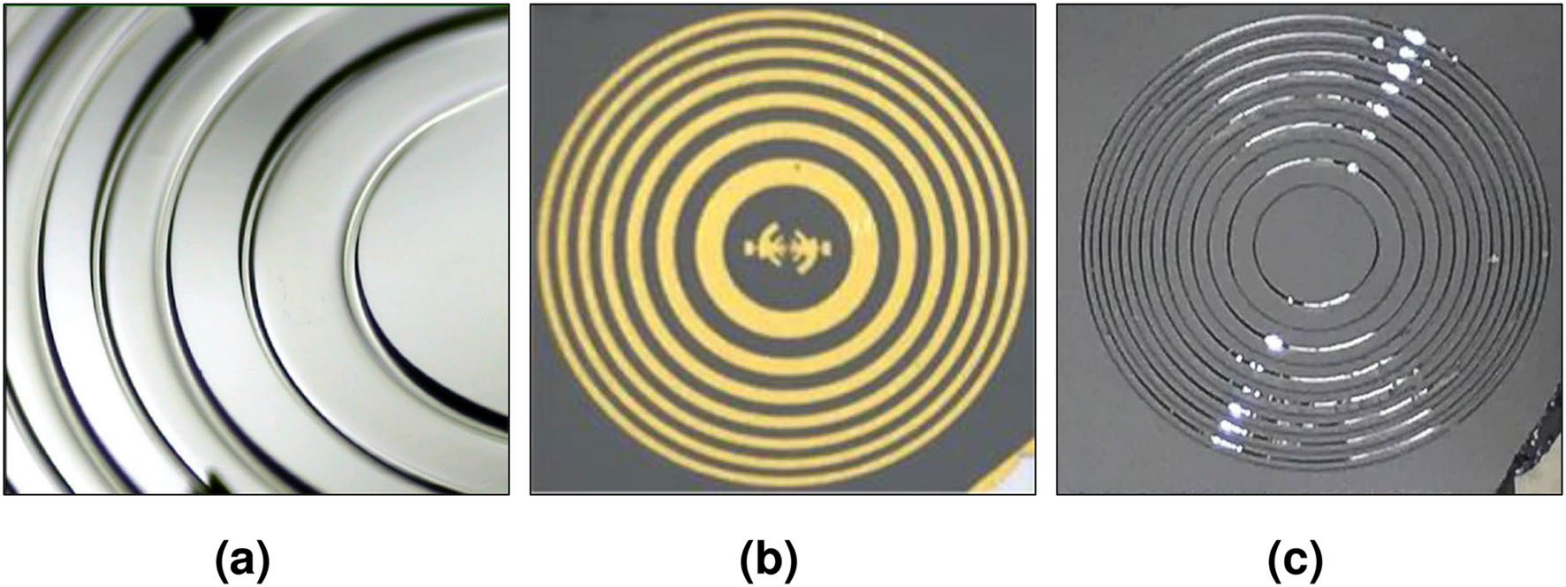
The optical image of the fabricated lens-antenna device: (**a**) a microscopic image of the edged FZP. (**b**) Log-periodic antenna side. (**c**) The FZP’s side.

However, the test samples show in Fig. 6a that the isotropic wet etching does not generate neither a homogenous surface nor a homogenous etching along the FZP.

The anisotropic etching has finally been chosen for the final device, due to the homogeneity which is given to the etched FZP. The results of the fabrication process can be seen in Fig. 6b and c, where the gold thickness of the Log-periodic antenna and the FZP was 150 nm and the final flat device is shown as a square of a size 9.41 mm.

## Measurement results

The new lens-antenna has been measured using a homodyne receiver and a Golay cell as a detector. In order to demonstrate the performance of the device, please check "Experimental setup" in the supplementary document for more information.

**Figure 7:**
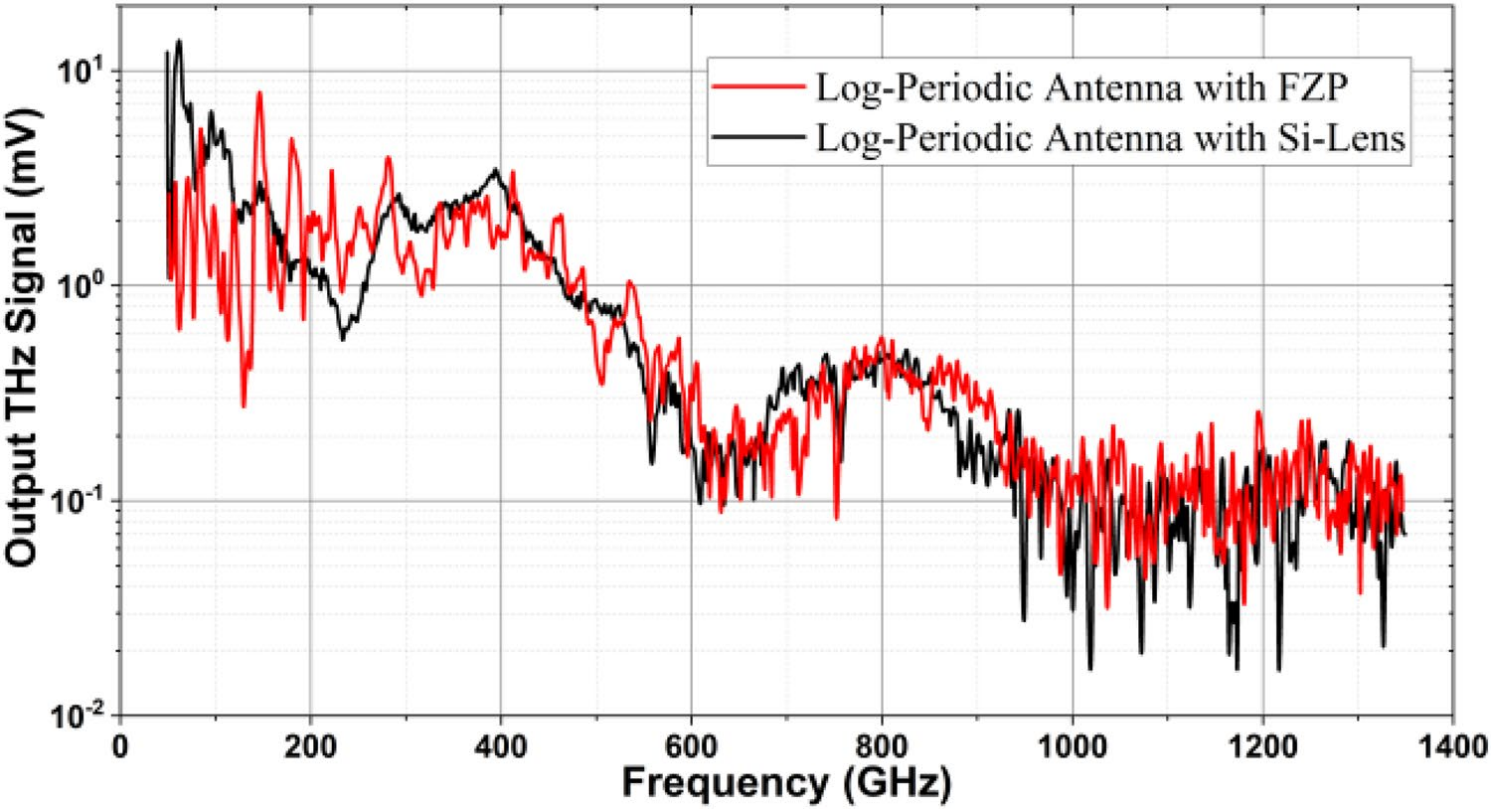
Comparison of the performance of the photomixer with an etching depth of 37 μm and the same photomixer with the Si-Lens.

The first device under test is the log-periodic antenna type, which is integrated with the lens-antenna that had an etched depth of 37 μm. Figure 7 shows the received THz power comparison between the lens-antenna with the standard Si-Lens and with the FZP (black and red curves, respectively). It is clear that the performance of the new integrated lens-antenna is perfectly comparable to the standard Si-Lens, especially at the frequency range around 1 THz. It has to be taken into account that the etching depth here is 5 μm below the optimum (42 μm). Thus, the expected performance at around 1 THz has been shifted to 800 GHz. This similarity in the performance of the integrated lens-antenna and the conventional method using the bulky Si-lens provides a huge step toward producing new types of sources and detectors on one chip and increases the compactness of the THz systems.

## Conclusion

The new integrated lens-antenna has a comparable performance compared to the bulky Si-lens, with a considerable reduction in size in all three dimensions, especially the lens thickness. Additionally, the new lens-antenna also presents many advantages over the bulky Si-lens like high directivity and less sensitivity to the THz source or detector misalignments. Moreover, the simple two-step lithography fabrication process makes it compact, monolithically integrable and less effected by misalignments. The versatility of this design will be suitable for nanophotomixers to improve the performance of THz system-on-chip.

## Supplementary Information

Supplementary Information.
