# Supplementary material for: Design and implementation of a terahertz lens-antenna for a photonic integrated circuits based THz systems
Source: Sci Rep. 2022 Jan 27;12:1476. doi: 10.1038/s41598-022-05338-0 (PMC8795237; doi:10.1038/s41598-022-05338-0)
Supplement: Supplementary file 1 — Supplementary Information. [file 41598_2022_5338_MOESM1_ESM.pdf]

Design and Implementation of a Terahertz Lens-Antenna for a Photonic Integrated Circuits Based THz Systems

(Supplementary document)

1. **Experimental setup.** Two slightly detuned DFB-LDs (wavelength ~ 850 nm) were used to generate the optical beat signal with a beating frequency corresponding to the frequency difference between the two DFB-LDs. The optical beat signal is used to illuminate the active area of the THz photomixer to generate THz waves. The THz waves were directed into the opening aperture of a Golay cell detector using two semi-parabolic mirrors. The use of the Golay cell detector is to transform the THz output signal into an electrical signal. Then, a lock-in amplifier was used to extract the amplitude of the signal in terms of voltage, as shown in Figures S1, S2, and S3.

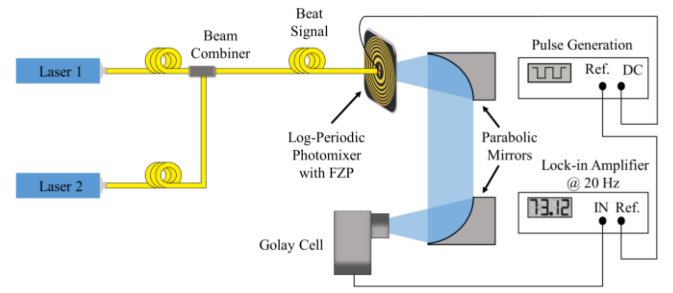

Figure S1: Schematic diagram of the THz setup.

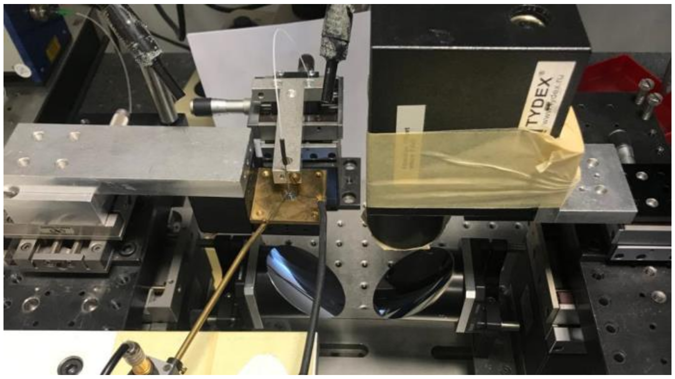

Figure S2: Optical image of the THz setup.

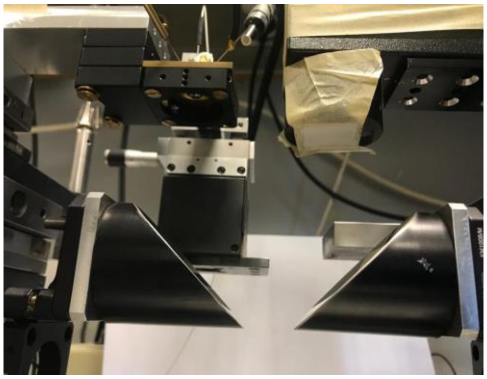

Figure S3: Side view of the THz setup.

2. Fresnel Zone Plate

Fresnel Zone Plates (FZP) work by interference or diffraction. An FZP is formed by concentric circular rings that alternate between opaque and transparent, placed on a transparent flat plate. The rings, or Fresnel zones, are separated in a way that the light that crosses the transparent zones interferes constructively at the focal point. This can be better appreciated in Figure S4, where the side and top views of an FZP are illustrated. Also, the normal use as a lens is seen here, where the source is placed at the focal point of the plate.

To derive the design equations of an FZP [1], let's consider a source on the same plane as the FZP but at a distance r from the center of it. The optical path length l between the source point and the focal point and the optical path length l0 between a source point at the center of the FZP and the focal point are given, respectively, in equations S1 and S2, and illustrated in Figure S5.

$$l = \sqrt{r^2 + f^2} \quad \text{(S1)}$$

$$l_o = f \quad \text{(S2)}$$

To locate source points that will interfere constructively at the focus it is required that the optical path lengths (l - l_0) differ by not more than $\lambda/2$:

$$(l - l_o) < \frac{\lambda}{2} \quad \text{(S3)}$$

Source points satisfying this criterion define the first Fresnel zone, i.e. the green area from Figure S6. As the source points move towards the outer part of the FZP, the distance to the center l-l_0 increases beyond $\lambda/2$. The sources with optical path lengths satisfying:

$$\frac{\lambda}{2} < (l - l_o) < \lambda \quad \text{(S4)}$$

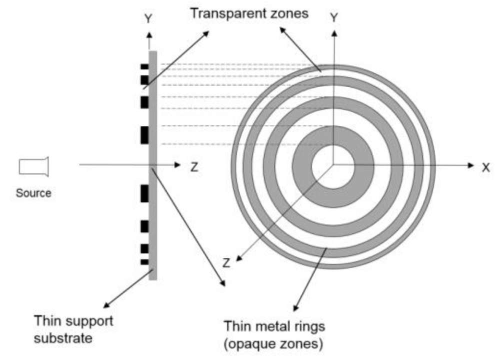

Figure S4: FZP (left) side view and (right) top view [1].

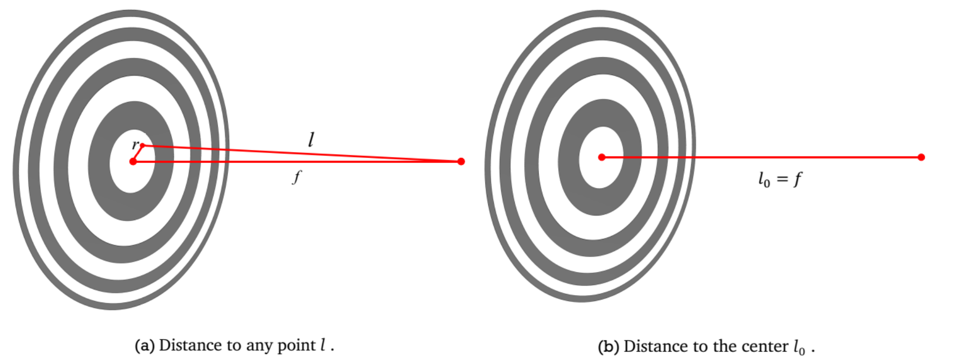

Figure S5: Distances from the focal point f to the FZP [1].

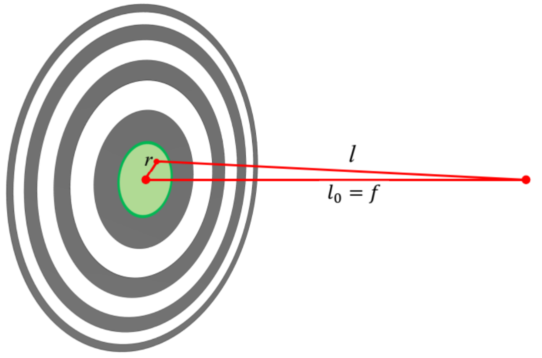

Figure S6: First Fresnel zone highlighted in green [1].

will be part of the second Fresnel zone (Figure S7) and will interfere destructively at the focus with the sources in the first zone. Generalizing this behavior to the *n*th zone, the source points in it will satisfy the following expression:

$$\frac{(n-1)\lambda}{2} < (l - l_o) < n\lambda \qquad \text{(S5)}$$

where *n* is an integer from *1* to *N*, where *N* is the total number of Fresnel zones in the plate. The odd zones of the plate will interfere constructively whereas the even zones will interfere destructively.

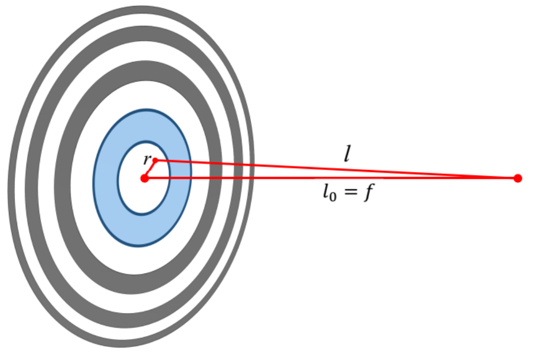

Figure S7: Second Fresnel zone highlighted in blue [1].

It can be noted that a FZP can start either with a transparent or an opaque zone and the effective lens will be the same. Therefore, optical path lengths differences satisfying the following expression will delimit the boundaries of the zones:

$$(l - l_o) < \frac{n\lambda}{2} \qquad \text{(S6)}$$

Rewriting the expression in terms of the source point's radii:

$$\sqrt{f^2 + r_n^2} - f = \frac{n\lambda}{2} \qquad \text{(S7)}$$

Solving equation S7 for r_n, the radii of the zones can be obtained by the expression in equation S8, where the wavelength λ and the focal distance f are taken into account.

$$r_n = n\lambda\left(f + \frac{n\lambda}{4}\right) \qquad \text{(S8)}$$

In order to calculate the zones of the FZP, the first step would consist of calculating the minimum number of zones required to satisfy the design specifications, which will include again the

frequency (or wavelength λ), the resolution (w) and the focal distance (f). All these parameters are considered in the following equation:

$$N = \frac{\lambda f}{4w^2} \quad \text{(S9)}$$

Taking a look into equations S8 and S9 it can be seen that the focal length of the FZP depends on the wavelength of radiation, whereas in the Fresnel lens it was independent of the frequency. However, it does not mean that the FZP is not working any more if a different frequency from the one used to design is radiated, but simply a change in the focal distance will be experienced. Another relation that can be extracted from these equations is that if resolution wants to be increased, then the number of zones has to be also higher. Therefore, a compromise between the size and the resolution must be taken.

Since half of the radiation is reflected backwards due to the opaque zones (metal rings), a big amount of energy does not propagate towards the focal plane. Thus, in comparison with the commonly used silicon lens, the efficiency becomes lower. Also, if the illuminating efficiency is low, i.e. the incident wave does not cover completely the plate, the focal point will be compromised. Although FZPs have some disadvantages compared with dielectric lenses, they appear to be a good solution in some X-ray applications like microscopy due to their flatness, fabrication simplicity and capability of high resolution focal points.

3. **Lens-Antenna Designs.** A log-periodic antenna was designed with the size ratio of tooth/anti-tooth = 0.707, the ratio of the radial sizes of successive teeth = 0.5, and the gap width between the antenna contacts = 10 mm [2]. Then, Ni (25 nm) and Au (150 nm) was evaporated on top of LTG-GaAs wafer (thickness ~ 350 mm) to form the log-periodic antenna using a standard optical lithography process. The parameters, such as the frequency, wavelength, Focal length, and resolution are chosen to be delimited from the whole THz range to be able to optimize the antenna or the resolution of the FZP. These general parameters are in Table S1. From the eq. S8 and eq. S9, The radii can be calculated in μm, as shown in Table S2.

Frequency	1 THz
Wavelength	03 mm
Focal length	15 mm
Resolution	150 μm

Table S1: General design parameters

r_1	1118.594	r_8	3179.178
r_2	1583.027	r_9	3374.341
r_3	1940.146	r_{10}	3559.306
r_4	2241.836	r_{11}	3735.586
r_5	2508.179	r_{12}	3904.360
r_6	2749.465	r_{13}	4066.560
r_7	2971.806	r_{14}	4222.945

Table S2. Radii of the FZP

4. **Implementation Process and Results.** A series of options for the THz antenna for a nanocontacts-based photomixer have been designed and simulated. Among all of them, one has been chosen to be implemented. The fabrication simplicity: the integrated FZP lens-antenna on LTG-GaAs

substrate with gold. The integrated FZP lens antenna was fabricated using standard optical lithography.

For the lithography process, two particular masks were chosen for the fabrication. A real picture of it is shown in Figure S8, where both designs can be seen. The circular shapes that do not have any antenna in the middle will correspond to the top FZP of the design, and therefore, the focusing element. Regarding the circular shapes that have an antenna, they correspond to the gold rings that are placed underneath the FZP. The log-periodic antenna, as well as the integrated photomixer, were employed. Moreover, some alignment marks are placed, delimiting a square around the FZPs to be able to align the top and bottom sides of the sample by dicing it. Therefore, the first step, after having cut and cleaned the LT-GaAs, will consist of, with the optical lithography, placing the FZP on the LT-GaAs. For this purpose, the photoresist AZ 1518 has been used for the lithography process. After that, the etching must take place. A depth of 41.65μm is needed, according to the calculation, therefore, since it is a high amount, wet etching has been chosen as the best option. To do so, a solution of water, sulfuric acid, and hydrogen peroxide, with the proportions of 100:10:80 (H_2O:H_2SO_2:H_2O_2), respectively, is employed. The resulting etching depth after 10 minutes in the solution has been 34 μm, i.e. 3.4 μm/min. This depth is not enough, thus, more samples will have to be implemented to optimize the etching time to reach the desired etching depth.

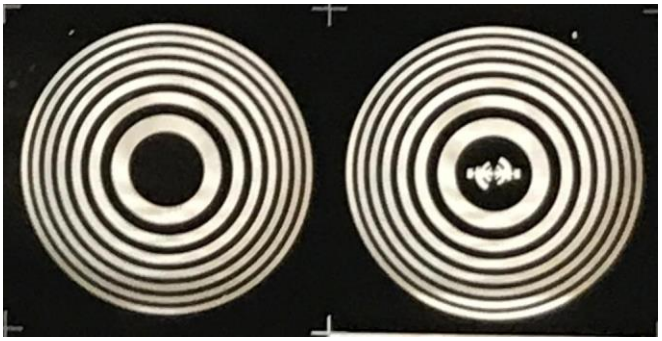

Figure S8: Top view of the fabricated mask.

The next step would be dicing the samples using the alignment marks mentioned before to align the top and bottom parts. On this bottom part, the gold rings and the integrated photomixer in the antenna will be placed, directly underneath the center of the first Fresnel zone. The same lithography process from before must be followed in this case again. The last step would be to evaporate gold to cover the antenna and the rings. Afterward, transversal cuts have been done to analyze the etched shape from the sides. The shape of the etching is crucial for the behavior of the implemented devices. In the case of the FZP, generating borders with some inclination will benefit the performance due to the nature of the diffractive lenses, for which smoother changes in phase will generate a better focal point.

References

1. "Fresnel zone plate theory." [Online]. Available: http://zoneplate.lbl.gov/theory.
2. R. Mendis, C. Sydlo, J. Sigmund, M. Feiginov, P. Meissner, and H. L. Hartnagel, "Tunable cw-thz system with a log-periodic photoconductive emitter," Solid-State Electron. 48(10-11), 2041–2045 (2004).
